# Call-Tracking Data and the Public Health Response to Bioterrorism-Related Anthrax

**DOI:** 10.3201/eid0810.020355

**Published:** 2002-10

**Authors:** Joshua A. Mott, Tracee A. Treadwell, Thomas W. Hennessy, Paula A. Rosenberg, Mitchell I. Wolfe, Clive M. Brown, Jay C. Butler

**Affiliations:** * Centers for Disease Control and Prevention, Atlanta, Georgia, USA; † Centers for Disease Control and Prevention, Anchorage, Alaska, USA

**Keywords:** anthrax, bioterrorism, triage, Centers for Disease Control and Prevention

## Abstract

After public notification of confirmed cases of bioterrorism-related anthrax, the Centers for Disease Control and Prevention’s Emergency Operations Center responded to 11,063 bioterrorism-related telephone calls from October 8 to November 11, 2001. Most calls were inquiries from the public about anthrax vaccines (58.4%), requests for general information on bioterrorism prevention (14.8%), and use of personal protective equipment (12.0%); 882 telephone calls (8.0%) were referred to the state liaison team for follow-up investigation. Of these, 226 (25.6%) included reports of either illness clinically confirmed to be compatible with anthrax or direct exposure to an environment known to be contaminated with *Bacillus anthracis*. The remaining 656 (74.4%) included no confirmed illness but reported exposures to “suspicious” packages or substances or the receipt of mail through a contaminated facility. Emergency response staff must handle high call volumes following suspected or actual bioterrorist attacks. Standardized health communication protocols that address contact with unknown substances, handling of suspicious mail, and clinical evaluation of suspected cases would allow more efficient follow-up investigations of clinically compatible cases in high-risk groups.

In response to the terrorist attacks on the World Trade Center and the Pentagon in the United States on September 11, 2001, preestablished emergency operations centers at the Centers for Disease Control and Prevention (CDC) were activated to assist in coordinating the public health response. After the first indication of a case of bioterrorism-related anthrax in Florida in October (1–4), the volume of calls to the emergency operations centers from the general public and health departments increased dramatically. In response to this increased demand, the preestablished centers were combined into an agencywide Emergency Operations Center (EOC), specialized teams were established to focus on specific local investigations, and staff was supplemented with additional personnel and resources.

A triage system was established to monitor incoming calls for referral to specialized teams (Figure 1). The State Liaison Team (SLT), which was established as a component of the second tier of this system, was formed to respond to calls from persons reporting illnesses and exposures possibly related to bioterrorism. The SLT assisted with the diagnostic evaluation of illness suspected of being due to anthrax exposure by collecting clinical data, providing information, interpreting recommendations, arranging for diagnostic testing or expert consultation, and facilitating case reporting with state and local health authorities. If highly suspicious illnesses warranted further epidemiologic investigations, the SLT assisted with referrals to field investigation or specialized teams. These teams then coordinated investigation activities with the appropriate state health departments (Figure 1).

**Figure 1:**
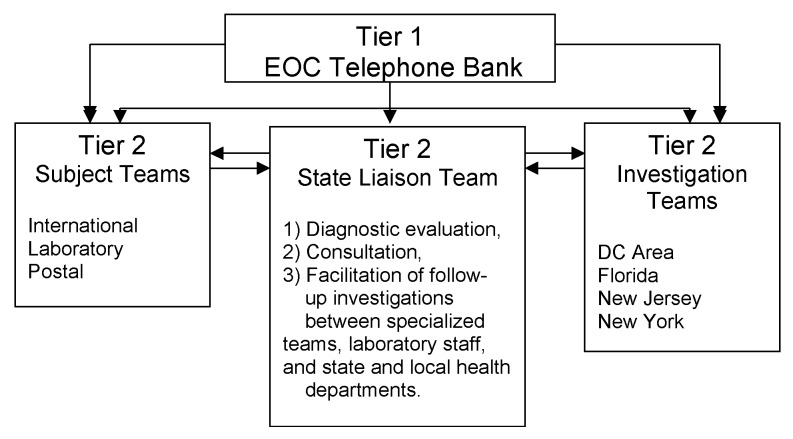
Emergency Operations Center (EOC) telephone call triage system, Washington, D.C., area, October 2001 through February 2002

We describe the nature and volume of telephone calls received by the EOC, as well as those referred specifically to the SLT for more detailed tracking and follow-up. We use the call data to highlight some implications for staffing strategies and to recommend changes in the EOC triage protocol that may allow second-tier referral teams to focus more exclusively on high-risk case investigations.

## Methods

A variety of professional staff screened calls coming into a central telephone bank. A prerecorded message instructed callers to contact their state or local health department if they had not done so. Calls that could be answered with “Frequently Asked Questions” documents or guidelines published in the Morbidity and Mortality Weekly Report (MMWR) were handled directly by public health advisors, epidemiologists, and junior staff (5–8). Callers were referred to the SLT for follow-up if they reported symptoms consistent with anthrax or other bioterrorism agents, noted exposure to a suspicious package or substance, or required detailed medical expertise. SLT staff included a team of public health advisors to obtain initial case information, and at least two physicians, epidemiologists, or veterinarians. The SLT had an average of nine staff members (range 2–15) that was reduced in evenings and on weekends depending on the volume of calls being received. Whenever appropriate, calls were also referred from the SLT to state epidemiologists for more detailed follow-up.

Two Access databases (Microsoft Corp., Redmond, WA) were created to assist in documenting and tracking all incoming calls. A general database was intended to document every incoming call to the EOC telephone bank. For all incoming calls—call volume permitting—central telephone bank staff were instructed to record information on the date, topic, and type of caller on call response forms. SLT staff regularly collected these forms for manual data entry. Reports of call volume, call type, and call topic by day were then shared with EOC management and communications personnel to assist them with staffing decisions, publication of MMWR reports, and determination of educational needs.

Calls referred from the central telephone bank to the SLT were manually entered into a second, more detailed SLT tracking database. Information collected in this tracking database included demographic background of the patient, reporter information, and any reported symptoms or exposures. SLT staff were also asked to assign each referred call to a risk category to prioritize follow-up within the large volume of calls.

Telephone call data were exported from Access databases into Statistical Analysis Software (SAS Institute, Inc., Cary, NC). Distributions of call volume by date of call, type of caller, and topic of call were produced from the central EOC telephone bank data. Descriptive analyses of SLT tracking data were undertaken by type of caller, state of reported occurrence, triage classification (level of urgency), reported signs and symptoms, and nature of reported exposure. Data were analyzed during the peak period of call volume during the anthrax investigations (October 8 to November 11, 2001).

## Results

### EOC Telephone Bank Data

From October 8 to November 11, 2001, a total of 11,063 telephone calls were documented and responded to by EOC telephone bank staff. A topic of call was indicated for 4,178 (37.8%) of the calls. The most frequently mentioned topic was “questions about the availability of an anthrax vaccine” (2,438 [58.4%] of 4,178 calls), followed by “request for general bioterrorism information” (617 calls [14.8%]), “request for information about personal protective equipment” (501 calls [12.0%]), “general concerns about bioterrorism” (491 calls [11.8%]), and “request for information about smallpox” (400 calls [9.6%]).1

The type of caller was indicated on 6,845 (61.9%) of the 11,063 call forms. The most frequent types of callers included private citizens (3,712 [54.2%] of 6,845 calls), followed by physicians (1,846 calls [27.0%]), other federal or state employees (714 calls [10.4%]), and nonphysician health-care professionals (672 calls [9.8%])^1^. A greater percentage of calls from private citizens (42.5%) than from health professionals (32.1%) mentioned concerns about smallpox, bioterrorism, or requests for bioterrorism information. Health professionals (2.7%) were more likely than private citizens (0.7%) to ask questions about sample handling and processing.

**Figure 2:**
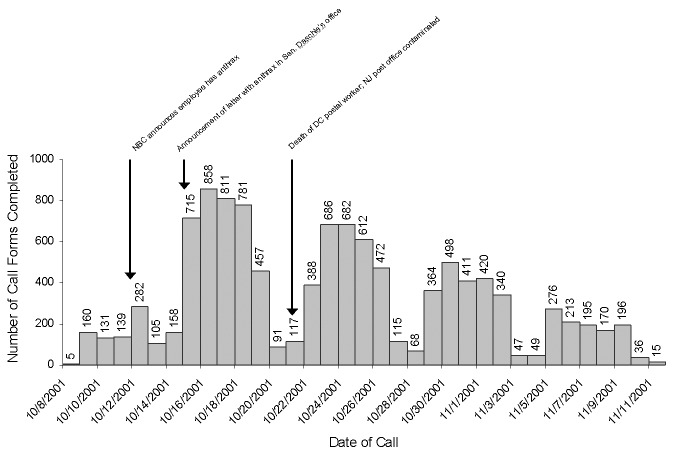
Telephone calls documented by staff of the Emergency Operations Center telephone bank, October 8 to November 11, 2001 (N = 11,063 call forms)

Call volume increased to a peak of 858 calls received on October 16, 2001, shortly after the public announcement that a letter containing anthrax had been opened in Senator Tom Daschle’s office (Figure 2). After that date, call volume to the EOC decreased each week. While the highly publicized nature of the bioterrorism-related events contributed to the large number of calls received by the EOC, day of the week was also an important determinant of call volume. Fewer calls were received on the weekends of October 13–14, 20–21, and 27–28 and November 3–4 and 10–11. During the period of data collection, the mean call volume to the EOC was 80 incoming calls per day on weekends and 411 incoming calls per day on weekdays. During weekdays, a lower call volume was also consistently observed on Mondays and Fridays. An average of 350 incoming calls per day were received on Mondays and Fridays and 450 incoming calls per day during Tuesday through Thursday. The proportion of calls received by topic of call and type of caller did not change in any meaningful way during this time (data not shown).

### SLT Follow-Up Tracking Data

Of the 11,063 calls received by the EOC telephone bank, 882 (8.0%) were referred to the SLT for follow-up. Calls referred to the SLT came most commonly from physicians (256 calls [29.0%]), followed by private citizens (178 calls [20.1%]); state health department employees (99 calls [11.2%]); local government, law enforcement, or emergency personnel (99 calls [11.2%]); and nonphysician health-care workers (82 calls [9.3%]). The type of caller was not documented for 168 (19.0%) of the calls referred to the SLT.

**Figure 3:**
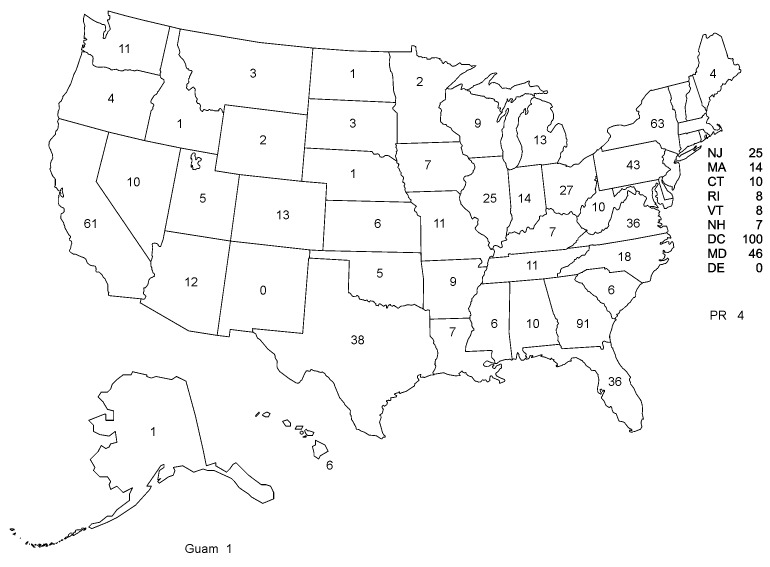
Distribution of telephone calls referred to the State Liaison Team, by state of occurrence, October 8 to November 11, 2001 (N = 882 calls)

The SLT staff provided follow-up on calls from 48 states, the District of Columbia, Puerto Rico, and Guam. Figure 3 presents the distribution of these calls by state of occurrence. While the distribution of calls by state was generally population based, a larger proportion of calls were received from states with increased press coverage of confirmed cases of anthrax and from Georgia, where CDC headquarters is located. Forty-six percent of SLT follow-up activities pertained to reported occurrences in Washington, D.C., Georgia, New York, California, Maryland, and Pennsylvania (Figure 3). The proportion of calls received from private citizens or physicians did not vary by region of the country (data not shown).

Because SLT staff was limited to an average of nine members, a triage protocol to classify calls referred to the SLT by level of urgency was developed (Table 1). In 10.4% of the calls referred to the SLT for follow-up, a physician or health-care professional reported symptoms clinically compatible with anthrax in a person from a known high-risk group (postal workers, U.S. government officials, national press from contaminated facilities, or person with known contact with a contaminated facility) or in a person who reported exposure to a suspicious substance. An additional 15.2% of calls referred to the SLT included a report by a health-care professional of a person with clinically compatible symptoms but no reported high-risk status or possible source of exposure. Forty-four percent of all calls referred to the SLT mentioned exposure to a suspicious package or substance but did not include any report of clinically confirmed signs or symptoms. An additional 30.4% of the calls referred to the SLT included no mention of any reported exposures, signs, or symptoms (Table 1).

**Table 1:** Telephone calls referred to Emergency Operations Center State Liaison Team (SLT), by risk category, October 8 to November 11, 2001

Risk/urgency classification	Criteria	Frequency (N = 882)	Percent (%) of all calls referred to SLT
Level 1: A “Confirmed”	A. Clinically compatible^a^ case *-and-* B. Isolation of *Bacillus anthracis* or two supportive lab results.	0^b^	0.0
B	A. Clinically compatible case *-and-* B. B. No isolation of *B. anthracis*, but one supportive lab result -*or-* epidemiologic link to confirmed exposure but no supportive lab results	2	0.2
C	A. Clinically compatible case –*and-* B. No epidemiologic link and no lab results *-and-* C. Known high-risk group: postal worker, U.S. government official, national press from contaminated facilities/or person with known contact with a contaminated facility *-or-* Ingestion of, inhalation of, or dermal contact with suspicious substance	45	5.1
D	A. No illness (or reports of symptoms that are clinically unconfirmed by a health professional) *-and-* B. Known direct exposure to environment confirmed to be contaminated with *B. anthracis*	45	5.1
E	A. Clinically compatible case *–and-* B. Not in high-risk group, *-and-* C. No lab results or epidemiologic link, *-and-* D. No known exposures to suspicious substance or packages	134	15.2
F	A. No illness (or reports of symptoms that are clinically unconfirmed by a health professional) -*and-* B. Not in high-risk group, *-and-* C. No lab results or epidemiologic link, *-and-* D. Ingestion of, inhalation of, or dermal contact with suspicious substance, or received mail directly from facility known to be contaminated during period of investigation.	388	44.0
G	A. No illness (or reports of symptoms that are clinically unconfirmed by a health professional) -*and-* B. Not in high-risk group, *-and-* C. No lab results or epidemiologic link, *-and-* D. No known exposure to suspicious powder or packages.	247	28.0
Unknown/not classified	A. Unknown or call not related to anthrax	21	2.4

^a^Clinically compatible refers to physician or health professional report of any symptom thought to be related to inhalational, cutaneous, or gastrointestinal anthrax. ^b^Cases of anthrax confirmed during this time period were identified through active surveillance by CDC field epidemiology teams and not the Emergency Operations Center telephone bank.

Of the 181 calls referred to SLT that mentioned signs or symptoms clinically compatible with anthrax (classified as level A, B, C, or E in Table 1), fever or influenzalike symptoms were most commonly reported (57 calls [31%]). Other commonly reported signs and symptoms included skin lesions or eschars (48 calls [26.5%]), upper respiratory symptoms (47 calls [26.0%]), and skin rashes (19 calls [10.5%]). Fewer calls included mention of sore throats (15 calls [8.3%]), myalgia (15 calls [8.3%]), gastrointestinal problems (8 calls [4.4%]), lymphadenopathy (6 calls [3.3%]), chest pain (6 calls [3.3%]), and shortness of breath (4 calls [2.2%])^2^.

Four hundred eighty calls (54.4%) referred to the SLT included mention of exposure to a suspicious substance or package or direct contact with an environment known to be contaminated with *B. anthracis* (classified as level B, C, D, or F in Table 1). Over half of reported exposures included mention of contact with a “suspicious” powder or package (Table 2). However, <10% of reported exposures (47/480) included mention of any clinically confirmed signs or symptoms compatible with anthrax. As a result, standardized response protocols to address the handling of suspicious packages and powders and the receipt of mail through contaminated facilities were developed (5,7). This measure allowed second-tier triage staff to devote more time to calls involving clinically compatible cases from high-risk groups and SLT medical staff to remain on-call at off-site locations during evenings and weekends.

**Table 2:** Nature of reported exposure reported in telephone calls referred to the State Liaison Team, October 8 to November 11, 2001

Reported exposure	No.	Percent (%)
Received letter or package with suspicious powder	181	37.7
Visited location where *Bacillus anthracis* was isolated	102	21.3
Unspecified exposure to suspicious powder	81	16.9
Received mail from mail facility where *B. anthracis* was isolated	57	11.9
Received suspicious package without powder	20	4.2
Other	38	7.9
Unknown	1	0.1
Total	480^a^	100

^a^ n = 480 calls, including a report of exposure.

None of the calls referred to the SLT were confirmed to be reports of cases of anthrax. The confirmed cases of anthrax were identified by the CDC field specialty teams or through calls made to the CDC director.

## Discussion

From October 8 to November 11, 2001, the EOC received 11,063 telephone calls pertaining to bioterrorism and referred 882 of these calls to the SLT for diagnostic evaluation, consultation, and coordination of follow-up activities. The volume of calls received during this time period demonstrated a considerable public need for guidance during this emergency.

Highly publicized incidents such as the opening of the letter in Sen. Daschle’s office were likely catalysts for the observed increases in call volume. However, day-to-day patterns in the call volume to the EOC telephone bank suggest that at predictable times during the week emergency staff resources can be relaxed. During the data collection period, the mean call volume to the EOC was 80% lower on weekends than on weekdays. Within the working week, mean call volumes were 23% lower on Mondays and Fridays than during the rest of the work week. As many staff worked 12–20 hour days during the height of this emergency, allowing staff to remain “on-call” at off-site locations on days of predictably lower call volume may help maintain staff morale and stamina through long periods of emergency center operations.

The EOC implemented a tiered telephone call triage system designed to allow highly suspicious cases and exposures to be tracked more closely by field epidemiology and specialty teams (Figure 1). Using scripted responses to frequently asked questions, this system effectively screened out many calls involving general queries about anthrax vaccines, requests for bioterrorism information, and the use of personal protective equipment. This approach allowed the SLT at the second tier of the triage system to spend more time interpreting clinically confirmed symptoms and laboratory results, and monitoring possible exposures for further referral to appropriate specialized teams.

These findings, however, also indicate that many calls received by the SLT did not pertain to known high-risk situations. During the data collection period, nearly 75% of calls referred to the SLT did not include a report of any clinically confirmed signs or symptoms or any direct contact with an environment known to be contaminated with *B. anthracis*. Of these calls, nearly 60% mentioned contact with a suspicious powder or package, but included no report of illness. As a result, to maintain specificity in tracking high-risk cases, scripted responses were developed to questions regarding 1) contact with unknown substances, 2) the receipt of mail through a facility that had been contaminated with *B. anthracis*, and 3) the report of clinically unconfirmed signs or symptoms (5,7). We recommend further refinement of these response protocols for inclusion in the first tier of the triage system, along with additional training of telephone bank staff in the overall objectives and methods of triage during bioterrorism emergencies. These measures would substantially reduce the call volume burden on second-tier staff and decrease the chance that a high-risk situation would be overlooked during a similar bioterrorism event.

State health departments typically expect that CDC will direct local calls back to them unless they have previously been referred to CDC (9). As more than half the calls to the EOC were from private citizens, a larger number of calls should also have been redirected from the EOC to appropriate contact persons at the state level (with minimal data entry and analysis by CDC). Such referrals would have allowed the EOC staff more time to respond to questions from physicians or health departments. The extent to which state and local health departments were satisfied with the assistance received from the EOC also remains unknown. A survey of state and local personnel who contacted the EOC system would assist CDC staff with quality improvement of the triage system and provide additional insight into the state perspective of appropriate respective roles during periods of emergency response.

These data have several limitations. An unknown number of calls to the EOC telephone bank were undocumented as first-tier staff were unable to complete all telephone call response forms during peak periods of call intensity. These high call volumes periodically resulted in delays in information transfer between tiers of the telephone call triaging system. In addition, the manual completion of telephone response forms resulted in a substantial amount of missing data, as first and second-tier EOC staff often overlooked key data elements in their efforts to provide timely responses to public demands. Several coding classification schemes on the telephone response forms also require revision. For example, we were unable retrospectively to determine the number of law enforcement or emergency medical service personnel who called the central phone bank or whether callers from state health departments were medical or public relations personnel.

Telephone-based hotlines underestimate the true number of cases of a disease and are dependent on media reports and general public interest (10). However, a telephone bank at CDC during an outbreak of hantavirus pulmonary syndrome identified 38% of confirmed cases (10). Computerization of the EOC triage system, including required fields for date and topic of call and type of caller would allow for timely transfer and analysis of complete and accurate telephone call data and perhaps provide a similar layer of passive surveillance for emerging bioterrorism events. However, the maintenance of such a system would require additional technical expertise in database development, management, and analysis (11). Medical expertise in first-tier telephone bank staff will continue to be needed to assure the accurate entry of data into any automated system.

Our findings suggest that available on-site staff resources can be adjusted to predictable daily patterns of call volume to increase long-term effectiveness and stamina during emergency periods. While the first tier of the EOC telephone call triage system effectively addressed a substantial portion of all incoming public inquiries during this emergency, standardized health communication protocols that address contact with suspicious substances, handling of suspicious mail, and the clinical evaluation of suspected cases in the absence and presence of confirmed exposure should also be added to first-tier response activities in a computerized triage system. This standardization would allow for a more effective triage system for inquiries and more efficient focus for follow-up investigations by specialized epidemiologic teams.

## Footnotes

1Percentages do not add up to 100% as a call could include more than one topic (e.g., requests for information about more than one topic) or type of caller (e.g., caller is a physician who works at a state health department).

2Percentages do not add up to 100% because callers often reported more than one sign or symptom.
